# Motives of Chinese foreign direct investment in Africa: With regulation effects of institutional quality

**DOI:** 10.1371/journal.pone.0326970

**Published:** 2025-06-25

**Authors:** Lilin Yuan, Peng Ji

**Affiliations:** 1 School of Economics and Management, Beijing University of Technology, Beijing, China; 2 School of Urban Economics and Management, Beijing University of Civil Engineering and Architecture, Beijing, China; South China Normal University School of Economics and Management, CHINA

## Abstract

This article analyzes the motives of Chinese foreign direct investment (FDI) in Africa and the impact of the institutional quality of the host country for the period between 2003 and 2022 from both static and dynamic perspectives by using the OLS, PPML, and GMM methods. The results show that: China has obvious market-, and efficiency-seeking motives, but weak resource- and strategic asset-seeking motives. There is a tipping point for market- and efficiency-seeking motives. Good institutional quality of the host country (region) facilitates FDI expansion. Investment inertia and factor endowments affect long-term FDI. We also examine the moderating effects of institutional quality and the Belt and Road Initiative (BRI) and found that: the host country’s (region’s) institutional environment optimization makes market size more attractive and labor costs less attractive. The BRI itself has no obvious influence on Chinese investment in Africa, while the impact of market size and labor costs became more significant after 2013. Hence, when selecting investment locations, Chinese enterprises should prioritize the host country’s (region’s) market size, labor costs, and institutional quality. Additionally, they should utilize the moderating effect of institutional quality to mitigate the disadvantages associated with higher labor costs.

## 1 Introduction

China’s foreign direct investment (FDI) has grown rapidly since 2003, and Chinese FDI in Africa has begun to take shape. Besides, Africa is one of the most important participants in the Belt and Road cooperation. The Belt and Road initiative (BRI) was proposed in 2013, bringing new opportunities for China’s investment in Africa. Whether the BRI has played a catalytic role in it?

The coronavirus disease (COVID-19) outbreak reduced global investment in Africa to $41.05 billion in 2020. However, it soared to $82.20 billion in 2021, accounting for approximately 10% of the global FDI in developing countries [1], indicating that Africa is still an important destination for FDI. Although the COVID-19 epidemic has affected global investment in Africa, China’s investment has remained relatively stable. China’s outward FDI stock in Africa had exceeded $42.00 billion and covered over 51 African countries by the end of 2023. Moreover, China’s FDI flows to Africa was $3.96 billion in 2023, growing at a rate of 118.80%, accounting for 7.52% of global FDI flows to Africa. Although China is a latecomer to investment in Africa, it has become the major source of FDI in the region, with around 3300 established Chinese enterprises [2].

Despite the efforts of African countries to improve their investment environment, the overall low institutional quality makes Africa still not the most attractive region for investment globally. Considering the above, we explore the following research questions. (1) What drives Chinese FDI in Africa? (2) Does Africa’s institutional quality affect Chinese FDI? How does institutional quality affect investment motives? (3) Have China’s motives for investing in Africa and the influence of institutional factors changed since the BRI was launched?

This study contributes to the literature on FDI in Africa in three ways. First, several studies have shown that market size, labor costs, natural resources, and institutional factors are important drivers of FDI inflows to Africa [3–10]. However, no consensus has been reached on the role of natural resources. Asiedu [11] found no significant relationship between natural resources and FDI in sub-Saharan Africa (SSA). Conversely, Soumaré et al. [12] showed that natural resources are key drivers of Chinese FDI in Africa. The West has often described China’s engagement with Africa as ‘new colonialism’ and viewed Chinese FDI in Africa as an attempt to obtain natural resources [13,14]. However, China’s share of FDI in Africa in comparison with that of other countries shows that China is not biased towards resource exploitation. Some studies have also shown that private Chinese multinational corporations tend to invest in countries with more manufacturing and service sectors than natural resources [15]. In addition, considering Africa’s economic development, strategic asset-seeking motives should be examined. Therefore, this study uses the latest panel data (2003–2022) on Chinese FDI in Africa to comprehensively capture the impact of market size, labor costs, natural resources, strategic assets, and institutional factors on FDI in Africa and identifies the main motives behind Chinese FDI in Africa. Moreover, this study examines whether the speculative motive has changed since the BRI was implemented.

Second, this study evaluates the impact of the institutional quality of the host country (region). Previous studies have analyzed the effects of institutional factors on FDI inflows to certain African countries from the perspectives of corruption and democracy [16,17]. This study uses the Worldwide Governance Indicators (WGI) to comprehensively analyze the multidimensional impact of institutional quality. In addition, this study examines the regulatory effect of institutional quality on main investment motives.

Finally, considering investment inertia, this study constructs both static and dynamic models to better identify the factors that influence Chinese FDI in Africa.

The remainder of this paper is organized as follows. Section 2 describes the details of Chinese FDI in Africa. Section 3 presents theories and hypotheses regarding the possible motives for Chinese FDI in Africa and the role of institutional quality in the host country (region). Section 4 develops the empirical models and explains all variables and data sources. Section 5 presents the estimation results and interpretation, along with mechanism checks. Section 6 summarises the main findings and concludes the paper.

## 2 Typical characterization

### 2.1 Annual trends

**Fig 1:**
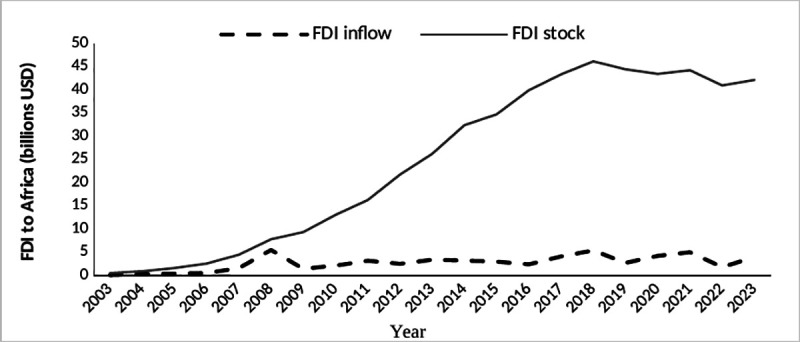
Chinese FDI outflows to Africa.

China is one of the main sources of inward FDI for African countries. As shown in Fig 1, Chinese FDI flow to Africa has been relatively stable, and the investment stock has grown steadily since 2009. Chinese FDI in Africa remains relatively steady despite the impact of the COVID-19. In 2023 Africa’s global FDI inward stock was $1036.25 billion, accounting for 2.11% of the global FDI inward stock [1]. Of this, Chinese FDI stock was $42.11 billion, accounting for 4.06% of the global FDI stock [2], with an average annual growth rate of 24.94%, compared with $490 million in 2003.

### 2.2 Regional characteristics

Table 1 illustrates the distribution of Chinese FDI stock and Chinese FDI as a percentage of GDP in African countries for the year 2022. Overall, Southeast African countries attract a relatively higher amount of Chinese FDI. Specifically, the countries with the highest investment stock are South Africa (ZAF) and the Democratic Republic of the Congo (COD). The geographic location of coastal countries can influences their ability to attract Chinese FDI. For instance, South Africa (ZAF), Nigeria (NGA), Algeria (DZA), and Kenya (KEN) have higher levels of FDI stock. However, some coastal countries, such as Namibia (NAM), Gabon (GAB), and Mauritania (MRT), represent the lower end of the FDI spectrum. Additionally, several landlocked or central countries, including Ethiopia (ETH), Niger (NER), and Zambia (ZMB), also show high levels of absorbed FDI stock.

**Table 1:** Chinese FDI stock and FDI/GDP for African countries in 2022.

ISO3 Code	FDI ($1M)	FDI/GDP (%)	ISO3 Code	FDI ($1M)	FDI/GDP (%)	ISO3 Code	FDI ($1M)	FDI/GDP (%)
AGO	194617	1.86	GHA	105826	1.43	NGA	232399	0.49
BDI	1959	0.59	GIN	104504	4.98	RWA	18188	1.37
BEN	16862	0.97	GMB	1997	0.92	SAH	–	–
BFA	664	0.04	GNB	2506	1.46	SDN	88595	1.71
BWA	14343	0.71	GNQ	23515	1.74	SEN	17681	0.64
CAF	958	0.40	KEN	178242	1.57	SLE	8750	2.14
CIV	80851	1.15	LBR	15578	3.89	SOM	–	–
CMR	38968	0.89	LBY	8754	0.16	SSD	5674	–
COD	412983	6.28	LSO	937	0.41	STP	51	0.09
COG	39522	2.50	MAR	28270	0.22	SYC	48614	23.62
COM	133	0.11	MDG	28194	1.84	TCD	56776	4.58
CPV	162	0.07	MLI	47803	2.55	TGO	5599	0.69
DJI	8582	2.34	MOZ	118035	6.41	TUN	2620	0.06
DZA	162192	0.72	MRT	18375	1.89	TZA	144082	1.90
EGY	120337	0.25	MUS	151566	11.72	UGA	69244	1.52
ERI	32043	4.39	MWI	19659	1.50	ZAF	574169	1.42
ETH	262032	2.07	NAM	17692	1.41	ZMB	197957	6.79
GAB	15216	0.72	NER	185356	12.01	ZWE	160485	5.86

For the distribution of Chinese FDI as a percentage of GDP, the data shows that Niger (NER) attracts a medium-high level of FDI, with FDI constituting the highest percentage of its GDP. Besides, Seychelles (SYC) and Mauritius (MUS) receive a low to medium level of investment, yet their FDI/GDP is also high. Only a few countries like Egypt (EGY) and Morocco (MAR) exhibit a relatively low share of Chinese FDI in relation to their GDP. Overall, Chinese FDI represents a significant portion of GDP for many African countries, highlighting the important role that Chinese FDI plays in the economic growth of African countries.

## 3 Theories and hypotheses

### 3.1 Investment motives

Dunning [18] classified FDI motives into four categories: market-seeking, natural-resource-seeking, efficiency-seeking, and strategic-asset-seeking. Based on Chinese data, Buckley et al. [19] confirmed that these four motives influence Chinese outward FDI. Early studies suggest that China has strategic asset-seeking motives for outward FDI in developed countries, but not in developing countries. However, with economic development catching pace in Africa, local enterprises have also increased factors such as their R&D input, marketing networks, and brand resources. In the light of the above, China’s motives for outward FDI in Africa is worth reassessing.

Market-seeking motive refers to FDI aimed at seeking emerging markets and expanding the market scale in the host country (region). Taylor [20] showed that the market size of the host country (region) is an important factor affecting the location choice for FDI. The potentially huge market size of African countries provides favourable conditions for FDI inflows from around the world. Claassen et al. [21] showed that Chinese investment in Africa targets growing markets that provide opportunities for Chinese firms to gain more experience in branding and managing their products. Folger [22] noted a significant association between market size and Chinese FDI inflows to SSA. Outward FDI has become one of the most effective and fastest ways obtain resources such as markets [23,24].

Resource-seeking motive pertains to FDI aimed at reducing production costs by utilising cheap foreign resources. Chen et al. [25] found that a country (region) with rich energy and mineral reserves can attract investments from multinational enterprises. Data show that a large proportion of the capital invested in Africa by various countries is concentrated in the energy and extractive industries [26]. Studies have also shown that oil and metal endowments have a positive and significant impact on FDI inflows to African countries (regions) [7,27,28]. In contrast, Chen et al. [29] found that Chinese FDI in Africa focuses more on market-seeking rather than resource-seeking motives.

Efficiency-seeking motive refers to FDI aimed at gaining economies of scale, transferring excess domestic production capacity to foreign countries, and improving the overall operating efficiency of enterprises by taking advantage of low labor costs in the host country (region) [30]. Cheap labor resources have always been one of the main factors determining FDI inflows. Okafor et al. [31] found that FDI in SSA is driven by efficiency-seeking motives. At the beginning of this century, Chinese firms did not have strong efficiency-seeking motives because of China’s labor endowment advantage. However, China’s demographic dividend has gradually disappeared in recent years and increased wage levels have diminished its labor cost advantage. Therefore, African countries with rich human resources have become important destinations for outward FDI from China.

Strategic-asset-seeking motive refers to FDI aimed at obtaining a host country’s technology, skills, and capabilities and strengthening investors’ market influence. Li and Fabus [32] argued that the strategic assets of a host country (region) are decisive factors affecting FDI inflows. The development of Africa increases the chances that investing firms will exploit African countries’ (region’s)  strategic assets, especially in emerging markets. Chang [33] found that patents have an insignificant effect on Chinese FDI in Africa.

Based on the theories above, this study proposes the following hypothesis:

**Hypothesis 1**: The larger the market size of African countries (regions), the richer the natural resources, the cheaper the labor costs, and the larger the reserves of strategic assets, the greater the FDI outflow from China to Africa.

### 3.2 Institutional factors

In addition to the four factors mentioned above, the impact of institutional quality on FDI in the host country (region) has received increasing attention. Some researchers found that the institutional quality of a host country (region) is positively correlated with FDI inflows [34–36]. However, the effect of institutional quality on FDI inflows in the developing country (region) has not yet been defined. Nkoa and Song [37], and Abor et al. [38] found that institutional quality increases the volatility of FDI inflows, suggesting that African authorities must reform their institutions. Na [39] used Eastern Africa as an example to demonstrate that building good institutions is important for attracting FDI. However, Buckley [27], Cheung and Qian [4], and Gao et al. [40] came to the opposite conclusion, namely, that FDI from China tends to flow into countries with high political risk.

Additionally, relevant literature has shown that institutional factors have different regulatory effects on different investment motives. Kolstad and Wiig [41] found that China is motivated to seek natural resources in host countries with imperfect institutions but not in those with perfect institutions. Wen and Yang [42] showed that China prefers host countries (regions) with higher institutional quality; however, this varies by country (region) and type of investors. They also found that different types of institutions can strengthen or weaken different motives. Therefore, we examine the impact of the institutional quality of host countries (regions) and analyze its regulatory effect on investment motives. Accordingly, we propose the second hypothesis:

**Hypothesis 2**: The institutional quality of host countries (regions) affects not only the scale of outward FDI from China to Africa but also investment motives. Moreover, the effects may differ based on the type of investment motives.

## 4 Model specifications and data

### 4.1 Model specifications

To explore the motives for Chinese FDI in Africa and the effects of institutional quality in host countries (regions), this study constructs the following model:


$$\begin{array}{c} \ln OFDI_{it}=\alpha_{0}+\alpha_{1}\ln GDP_{it}+\alpha_{2}\ln PGDP_{it}+\alpha_{3}\ln Res_{it}+\alpha_{4}\ln As_{it}+\alpha_{5}WGI_{it}\\ \;+\beta_{1}\ln GDP_{it}\times WGI_{it}+\beta_{2}\ln PGDP_{it}\times WGI_{it}+\beta_{3}\ln Res_{it}\times WGI_{it}\\ \;+\beta_{4}\ln As_{it}\times WGI_{it}+\gamma X_{it}+\eta_{t}+\varepsilon_{it} \end{array}$$ (1)

Accounting for inertia in investment, we establish the following dynamic model:


$$\begin{array}{c} \ln OFDI_{it}=\alpha_{0}+\alpha_{1}\ln OFDI_{i,t-1}+\alpha_{2}\ln GDP_{it}+\alpha_{3}\ln PGDP_{it}+\alpha_{4}\ln Res_{it}+\alpha_{5}\ln As_{it}\\ \;+\alpha_{6}WGI_{it}+\beta_{1}\ln GDP_{it}\times WGI_{it}+\beta_{2}\ln PGDP_{it}\times WGI_{it}+\beta_{3}\ln Res_{it}\times WGI_{it}\\ \;+\beta_{4}\ln As_{it}\times WGI_{it}+\gamma X_{it}+\eta_{t}+\varepsilon_{it} \end{array}$$ (2)

Here, $\ln OFDI_{it}$ is the explained variable, which represents Chinese FDI stock in country (region) *i* of Africa in year *t*; $\ln OFDI_{i,t-1}$ is FDI stock lagged by one year; $\ln GDP_{it}$, $\ln PGDP_{it}$, $\ln Res_{it}$, and $\ln As_{it}$ refer to total GDP, GDP per capita, natural resource endowment, and total strategic assets of country (region) *i* in year *t*, respectively (which indicate market-seeking, efficiency-seeking, resource-seeking, and strategic-assets-seeking motives, respectively); $WGI_{it}$ refers to the institutional quality of country (region) *i* in year *t*; $X_{it}$ are control variables, $\eta_{t}$ is a time fixed effect, and$\varepsilon_{it}$ is a random disturbance term.

### 4.2 Data description and variable selection

Considering the availability of data, this study restricts the research period to 2003–2022. This study uses data from the ‘Statistical Bulletin of China’s Outward Foreign Direct Investment’ (2004–2023), the World Bank database, the official website of the Ministry of Commerce of the People’s Republic of China, CEPII database, International Energy Agency and Li et al. [43].

(1) **Explained variable**

The scale of outward FDI from China to Africa is indicated by the FDI stock. Compared with FDI flows, FDI stock is a better indicator of the FDI scale in host countries (regions), as the latter avoids the problems of negative values and large short-term fluctuations associated with data on FDI flows.

(2) **Investment motives**

Following existing research [27,41,42,44], this study uses GDP to represent market size; GDP per capita to represent labor costs; the total energy production to represent natural resource endowments; and the proportion of high-tech product exports in total exports to represent the abundance of strategic assets.

(3) **Institutional quality**

Institutional quality is indicated by the Worldwide Governance Indicators (WGI), a system that includes six indicators: Voice and Accountability, Political Stability and Absence of Violence/Terrorism, Government Effectiveness, Regulatory Quality, Rule of Law, and Control of Corruption. The value of each of these six indicators ranges from −2.5 to 2.5. The higher the value, the better the institutional quality. We add 2.5 to each index to make the index non-negative and facilitate the interpretation of the results. Next, we sum the scores of the six indicators to obtain the total quality level.

(4) **Control variables**

The control variables include openness, the proportion of total goods and services exports in the GDP of the host country (region), and China’s overseas industrial parks. The variable ‘bilateral investment agreements’ equals 1 if the host country (region) has signed an agreement with the home country, and 0 otherwise. The variable ‘China’s overseas industrial parks’ takes the value 1 if the host country (region) has a Chinese overseas industrial park, and 0 otherwise.

Table 2 reports the statistical description of the above variables.

**Table 2:** Statistical description.

Variable	Symbol	Mean	Variance	Min	Max	Data source
FDI	$\ln OFDI_{it}$	8.6131	2.9224	0.0000	13.5242	Statistical Bulletin of China’s Outward Foreign Direct Investment
GDP	$\ln GDP_{it}$	23.1714	1.6167	18.4413	27.0762	World Bank
GDP per capita	$\ln PGDP_{it}$	7.2096	1.0484	3.4515	9.8960	World Bank
Natural resource	$Res_{it}$	1.1272	1.5643	0.0000	5.2362	International Energy Agency
Strategy asset	$As_{it}$	0.0980	0.3040	0.0000	4.5849	World Bank
Institutional quality	$WGI_{it}$	10.9875	3.6136	2.3175	20.2219	World Bank
Openness	$open_{it}$	3.2874	0.7806	0.0000	5.1358	World Bank
Bilateral investment agreements	$BIT_{it}$	0.3375	0.4731	0.0000	1.0000	Ministry of Commerce of the People’s Republic of China
Overseas industrial parks	$sez_{it}$	0.2490	0.4327	0.0000	1.0000	Li et al. (2019)

(1) For *BIT*_*it*_,we set the signing year of the agreement as 1. (2) For *sez*_*it*_, we search websites provided by Li et al. (2019) to supplement data of 2019–2022.

## 5 Main results and mechanism checks

Given that the FDI stock data of some countries are 0, to avoid the possible heteroscedasticity problem, this study uses both the ordinary least squares (OLS) and Poisson pseudo-maximum likelihood (PPML) methods. The PPML method assigns the same weight to all observations [45,46], thereby improving the heteroscedasticity problem caused by alternative investment amounts.

### 5.1 Benchmark regression

Table 3 reports the estimation results of the benchmark regression. Models (1) to (3) use the OLS estimation method. The results of the White test show P-values of less than 0.01 for Models (1) and (2). Thus, it can be concluded that the OLS estimation is biased, and the PPML method is chosen based on existing studies. Models (4) to (6) use the PPML estimation method. Model (4) considers the impact of investment motives and institutional quality, Model (5) adds control variables, and Model (6) further controls for the time effect. The mean VIF of the model is 1.74, and all individual VIF are less than 3, which indicates that the model does not have serious multicollinearity problems.

**Table 3:** Benchmark regression.

Model	(1)	(2)	(3)	(4)	(5)	(6)
Methods	OLS	OLS	OLS	PPML	PPML	PPML
Variables	$\ln OFDI_{it}$	$\ln OFDI_{it}$	$\ln OFDI_{it}$	$OFDI_{it}$	$OFDI_{it}$	$OFDI_{it}$
$\ln GDP_{it}$	1.2578***	1.0249***	0.7389***	0.7894***	0.6900***	0.6105***
	(0.0737)	(0.0754)	(0.0620)	(0.0432)	(0.0622)	(0.0576)
$\ln PGDP_{it}$	0.2859**	0.2625**	−0.3704***	−0.1218**	−0.1273*	−0.4268***
	(0.1188)	(0.1059)	(0.0955)	(0.0567)	(0.0764)	(0.0646)
$Res_{it}$	−0.3015***	−0.3616***	0.0833	−0.0729*	−0.1337***	0.0349
	(0.1057)	(0.0921)	(0.0773)	(0.0428)	(0.0425)	(0.0400)
$As_{it}$	−0.9101***	−0.6404***	0.1374	−1.2161***	−0.4604**	−0.1511
	(0.2672)	(0.2285)	(0.1696)	(0.3351)	(0.2142)	(0.2853)
$WGI_{it}$	−0.0631*	−0.0730**	0.0343	0.0217	0.0067	0.0379**
	(0.0325)	(0.0298)	(0.0270)	(0.0195)	(0.0190)	(0.0155)
$open_{it}$		0.3617***	0.6428***		0.3771***	0.5718***
		(0.0854)	(0.0657)		(0.1331)	(0.1215)
$BIT_{it}$		0.4364***	0.5402***		0.2887***	0.3937***
		(0.1453)	(0.1163)		(0.1114)	(0.0901)
$sez_{it}$		1.9324***	1.0963***		0.9574***	0.4774***
		(0.1207)	(0.1152)		(0.1241)	(0.1216)
Cons	−21.4647***	−17.5684***	−12.2479***	−7.3719***	−6.5360***	−3.2588**
	(1.9357)	(1.8987)	(1.5221)	(1.0114)	(1.4158)	(1.3966)
Time effect	No	No	Yes	No	No	Yes
obs	1,039	1,039	1,039	1,039	1,039	1,039
R^2^	0.4125	0.4884	0.6429	0.3648	0.4279	0.5996

The asterisks ***, **, and * indicate levels of signifificance at 1%, 5%, and 10%, respectively.

Model (6) shows that the estimated coefficient of $\ln GDP_{it}$ is 0.6105 and significantly positive, indicating that a 1% increase in the GDP of the host country (region) results in a 0.6105% increase in China’s outward FDI in Africa. This demonstrates a market-seeking motive for investment. The estimated coefficient of $\ln PGDP_{it}$ is 0.4268 and significantly negative, which signifies that a 1% decrease in labor costs corresponds to a 0.4268% increase in Chinese FDI in Africa, reflecting an efficiency-seeking motive. The estimated coefficient of $Res_{it}$ and $As_{it}$ is not significantly, indicating that China has no obvious resource- and strategic-asset-seeking motive in Africa. As African economies grow, the significance of energy resources diminishes, while the attractiveness of larger markets and cost-competitive labor increases. Nevertheless, there is still a need to improve the quality of Africa’s strategic assets.

The estimated coefficient of $WGI_{it}$ is 0.0379 and significantly positive, indicating that each unit increase in WGI is associated with a 0.0379% increase in Chinese FDI in Africa. This means that the better the institutional quality of the host country (region), the lower the investment risk, and the greater the potential to attract FDI.

The estimated coefficients of the other control variables are in line with expectations. The estimated signs of $open_{it}$ are significantly positive, indicating that complementary relationship between export and inward FDI for African countries (regions). The estimated sign of $BIT_{it}$ is significantly positive, indicating that bilateral investment agreements can protect the interests of Chinese investors to a certain extent, thereby promoting FDI in Africa. The estimated sign of $sez_{it}$ is significantly positive, indicating that the establishment of overseas industrial parks can not only provide preferential and convenient conditions for enterprises settled in the parks but also have scale effects for enterprises, improving their operating income and ultimately prompting them to expand investment.

Overall, Chinese FDI in Africa has clear market-, and efficiency-seeking motives, while the resource-, and strategic-asset-seeking motives are not obvious. Good institutional quality remains crucial for improving the prospects of FDI from China.

### 5.2 Robustness test

To verify the robustness of the model, we added a dummy variable to indicate country’s location, excluded the effects of the financial crisis and epidemic year, and apply 1% Winsorization. The results are shown in Table 4. Model (1) includes a dummy variable indicating whether a country is coastal. Models (2) and (3) show the estimation results after excluding the years 2008–2009 and 2020 –2022, respectively. Model (4) accounts for the removal of both impact years. Model (5) applies 1% Winsorization to the data. Model (6) controls country fixed effect. The findings from Models (1) to (6) indicate that Chinese FDI in Africa continues to be driven primarily by significant market- and efficiency-seeking motives. The effect of institutional quality is also positive. The results are generally consistent with the benchmark estimation results, indicating that the model setting is robust. Model (7) further examines the effects of squared term of the motives. It reveals that there is a tipping point for market- and efficiency-seeking motives. Specifically, Chinese FDI in Africa decreases when either the host country’s market size or labor costs exceed this tipping point.

**Table 4:** Robustness test.

Model	(1)	(2)	(3)	(4)	(5)	(6)	(7)
Methods	PPML	PPML	PPML	PPML	PPML	PPML	PPML
Variables	$OFDI_{it}$	$OFDI_{it}$	$OFDI_{it}$	$OFDI_{it}$	$OFDI_{it}$	$OFDI_{it}$	$OFDI_{it}$
$\ln GDP_{it}$	0.6225***	0.6161***	0.5769***	0.5850***	0.6131***	2.1173***	4.4856***
	(0.0602)	(0.0594)	(0.0683)	(0.0718)	(0.0558)	(0.4737)	(0.8442)
$\ln PGDP_{it}$	−0.4017***	−0.4249***	−0.4049***	−0.4037***	−0.4173***	−1.6160***	−3.6512***
	(0.0700)	(0.0648)	(0.0756)	(0.0762)	(0.0629)	(0.4702)	(0.5561)
$Res_{it}$	0.0544	0.0213	0.0667	0.0498	0.0194	0.2267***	0.1853**
	(0.0412)	(0.0405)	(0.0480)	(0.0493)	(0.0399)	(0.0846)	(0.0760)
$As_{it}$	−0.1546	−0.1766	0.0132	−0.0103	−0.3784	−0.9288***	0.4895
	(0.2819)	(0.3059)	(0.2587)	(0.2772)	(0.3224)	(0.1772)	(1.0726)
$WGI_{it}$	0.0382**	0.0317**	0.0455**	0.0375**	0.0285*	0.0599**	0.0437***
	(0.0150)	(0.0154)	(0.0180)	(0.0182)	(0.0153)	(0.0240)	(0.0150)
$\ln GD{P_{it}}^{2}$							−0.0786***
							(0.0180)
$\ln PGD{P_{it}}^{2}$							0.2138***
							(0.0353)
$Re{s_{it}}^{2}$							−0.0225
							(0.0160)
$A{s_{it}}^{2}$							−1.8275
							(2.3288)
Cons	−3.6474**	−3.4571**	−2.4461	−2.7425	−3.1965**	−29.5012***	−38.6884***
	(1.4411)	(1.4501)	(1.6401)	(1.7362)	(1.3594)	(8.1635)	(10.0580)
Control variables	Yes	Yes	Yes	Yes	Yes	Yes	Yes
Time effect	Yes	Yes	Yes	Yes	Yes	Yes	Yes
Country effect	No	No	No	No	No	Yes	No
obs	1,039	935	884	780	1,039	1,039	1,039
R^2^	0.6176	0.5994	0.6057	0.6058	0.5904		0.6684

The asterisks ***, **, and * indicate levels of signifificance at 1%, 5%, and 10%, respectively.

### 5.3 Endogeneity test

Although some control variables and year fixed effects are included in the model to eliminate endogeneity problems caused by omitted variables as much as possible, the reciprocal causality between FDI stock and investment motives may also contribute to the endogeneity problem. Therefore, this study utilises the first-order lagged terms of$\ln GDP_{it}$, $\ln PGDP_{it}$, $Res_{it}$, and $As_{it}$ as instrumental variables, as estimated using two-stage least squares (2SLS) and PPML regression methods, respectively. The results are shown in columns (1) and (2) of Table 5. According to the results, Chinese FDI in Africa remains driven by significant market-, and efficiency-seeking motives after endogeneity issues are considered. The effect of institutional quality is positive.

**Table 5:** Endogeneity test.

Model	(1)	(2)	(3)	(4)
Methods	2SLS	PPML	System GMM	Difference GMM
Variables	$\ln OFDI_{it}$	$OFDI_{it}$	$\ln OFDI_{it}$	$\ln OFDI_{it}$
$\ln OFDI_{i,t-1}$			0.7266***	0.2960***
			(0.0454)	(0.0752)
$\ln GDP_{it}$	0.6900***	0.6081***	0.1988**	3.1120**
	(0.0545)	(0.0580)	(0.0786)	(1.5575)
$\ln PGDP_{it}$	−0.4056***	−0.4360***	−0.3505***	−4.0729**
	(0.0861)	(0.0657)	(0.0910)	(1.9403)
$Res_{it}$	0.1220*	0.0370	0.2006*	0.4991**
	(0.0639)	(0.0409)	(0.1083)	(0.2030)
$As_{it}$	0.1781	−0.0219	−0.8482**	−0.3327*
	(0.2496)	(0.2279)	(0.3644)	(0.1931)
$WGI_{it}$	0.0306	0.0376**	0.0563***	0.1024
	(0.0200)	(0.0155)	(0.0182)	(0.1901)
Cons	−10.4794***	−2.9137**	−0.9463	
	(1.2837)	(1.3808)	(1.5708)	
AR(1)			−3.00	−2.63
P-value			(0.003)	(0.008)
AR(2)			−1.12	−1.19
P-value			(0.263)	(0.233)
Sargan test			87.50	104.98
P-value			(0.375)	(0.272)
Control variables	Yes	Yes	Yes	Yes
Time effect	Yes	Yes	Yes	Yes
obs	987	988	987	935
R^2^	0.6219	0.5973		

The asterisks ***, **, and * indicate levels of signifificance at 1%, 5%, and 10%, respectively.

In addition, considering that investment is a long-term behavior subject to inertia, China’s early investment scale in Africa would also impact current investment decisions. Models (3) and (4) show the results of the System Generalised Method of Moments (GMM) and Difference GMM, respectively. To some extent, dynamic GMM modelling can also eliminate the endogeneity problem resulting from mutual causation between variables.

Model (3) shows that the estimated coefficient of $\ln OFDI_{i,t-1}$ is 0.7266 and statistically significant, indicating that investment in the previous period has a significant and large impact on current investment. Given the relatively high coefficient for $\ln OFDI_{i,t-1}$, we conducted a multicollinearity test. The results indicated a Mean VIF value of 1.88, with all individual VIF values below 3, suggesting that the model does not exhibit significant multicollinearity issues.

Under dynamic conditions, the signs of the coefficients of $\ln GDP_{it}$ and $\ln PGDP_{it}$ representing investment motives remain unchanged. This demonstrates that China has clear market-, and efficiency-seeking motives in Africa.

Model (4) further reveals that while resource-seeking motive plays a significant role in China’s engagement with Africa, market size and labor costs are the primary factors driving Chinese investment decisions in the region. The estimated coefficient of $As_{it}$ is significantly negative, indicating that as the reserves of strategic assets in Africa increase, China’s FDI decreases. This suggests that China does not have a strong motivation to pursue strategic assets in the region.

The estimated coefficient of $WGI_{it}$ is no longer significant. One possible reason could be investment inertia; existing investment behaviour cannot be reversed in the short term, even if a sudden change in the political situation of the host country (region) leads to a decline in its institutional quality.

### 5.4 Mechanism analysis

(1) **Institutional quality**

In addition to directly affecting the amount of investment in the home country, the host country’s (region’s) institutional quality indirectly affects the amount of investment in the home country through its moderating effect on incentives to invest [42]. Table 6 reports the moderating effect of the host country’s (region’s) institutional quality on investment incentives. These results indicate that institutional quality has a significant regulatory effect on investment motives. With the optimization of the institutional environment in the host country (region), market size becomes more attractive, while labor costs, natural resources, and strategic assets become less attractive. This is because labor costs are low in most parts of Africa; therefore, investing firms care more about the political stability of the host country (region) to better secure their investment than about obtaining cheap labor. While natural resources and strategic assets are not primary investment factors, improving institutional quality will likely diminish their importance in investment decisions.

**Table 6:** Regulation effects of institutional quality.

Model	(1)	(2)	(3)	(4)
Methods	PPML	PPML	PPML	PPML
Variables	$OFDI_{it}$	$OFDI_{it}$	$OFDI_{it}$	$OFDI_{it}$
$\ln GDP_{it}$	0.3004***	0.7356***	0.6102***	0.6072***
	(0.1022)	(0.0515)	(0.0523)	(0.0574)
$\ln PGDP_{it}$	−0.3868***	−1.3770***	−0.2989***	−0.4379***
	(0.0711)	(0.1335)	(0.0637)	(0.0658)
$Res_{it}$	0.0109	0.0072	−0.5111***	0.0296
	(0.0363)	(0.0364)	(0.0734)	(0.0388)
$As_{it}$	−0.7837**	−0.8975***	−1.4271***	−3.4353*
	(0.3695)	(0.3023)	(0.4294)	(2.0504)
$WGI_{it}$	−0.6938***	−0.6688***	−0.0688***	0.0248*
	(0.1931)	(0.0764)	(0.0196)	(0.0150)
$\ln GDP_{it}\times WGI_{it}$	0.0299***			
	(0.0081)			
$\ln PGDP_{it}\times WGI_{it}$		0.0901***		
		(0.0099)		
$Res_{it}\times WGI_{it}$			0.0465***	
			(0.0063)	
$As_{it}\times WGI_{it}$				0.2597*
				(0.1445)
Cons	4.2777*	1.4578	−2.5290**	−2.8921**
	(2.4870)	(1.5454)	(1.2020)	(1.3822)
Control variables	Yes	Yes	Yes	Yes
Time effect	Yes	Yes	Yes	Yes
Obs	1,039	1,039	1,039	1,039
R^2^	0.6500	0.7040	0.7141	0.6206

The asterisks ***, **, and * indicate levels of signifificance at 1%, 5%, and 10%, respectively. The dynamic models pass AR(1), AR(2) and Sargan tests.

Investment inertia is a significant factor influencing Chinese FDI allocation in Africa. To analyze the potential impact of this inertia on the moderating effects of institutional quality across various investment motives, we employ a Difference GMM approach. Table 7 presents empirical findings, which reveal that improved institutional quality in host countries (regions) has notable moderating effects under dynamic conditions. Specifically, it enhances the market-seeking motive while simultaneously reducing the efficiency-seeking motive. However, there is no statistically significant moderating influence on resource- and strategic asset-seeking motives. These results suggest that market size and labor costs are the primary drivers of China’s investment strategy in Africa, with institutional quality mainly affecting these two factors.

**Table 7:** Regulation effects of institutional quality in dynamic conditions.

Model	(1)	(2)	(3)	(4)
Methods	Difference GMM	Difference GMM	Difference GMM	Difference GMM
Variables	$\ln OFDI_{it}$	$\ln OFDI_{it}$	$\ln OFDI_{it}$	$\ln OFDI_{it}$
$\ln OFDI_{i,t-1}$	0.2321***	0.2312***	0.2764***	0.2801***
	(0.0749)	(0.0830)	(0.0804)	(0.0713)
$\ln GDP_{it}$	3.0299	4.2981**	2.7525*	3.0309*
	(2.1529)	(2.1012)	(1.4733)	(1.6492)
$\ln PGDP_{it}$	−5.0157**	−6.2545**	−3.7083**	−4.0277**
	(2.5233)	(2.7599)	(1.8186)	(2.0009)
$Res_{it}$	0.2711	0.3135	−0.2944	0.5153***
	(0.2595)	(0.2435)	(1.0149)	(0.1915)
$As_{it}$	−0.3531**	−0.3496**	−0.3517**	−0.9412
	(0.1534)	(0.1545)	(0.1756)	(1.0763)
$WGI_{it}$	−3.7296**	−1.0102	−0.0109	0.0590
	(1.8017)	(0.6730)	(0.2082)	(0.1883)
$\ln GDP_{it}\times WGI_{it}$	0.1697**			
	(0.0802)			
$\ln PGDP_{it}\times WGI_{it}$		0.1611*		
		(0.0976)		
$Res_{it}\times WGI_{it}$			0.0896	
			(0.1230)	
$As_{it}\times WGI_{it}$				0.0906
				(0.1515)
Control variables	Yes	Yes	Yes	Yes
Time effect	Yes	Yes	Yes	Yes
Obs	935	935	935	935

The asterisks ***, **, and * indicate levels of signifificance at 1%, 5%, and 10%, respectively.

(2) **Effect of the Belt and Road Initiative**

The study also analyses the possible impact of the BRI, and the results are shown in Table 8. Models (1) and (2) consider differences in China’s motives driving FDI in Africa before and after the BRI. The results show that, after the BRI, the market- and efficiency-seeking motives driving Chinese FDI in Africa increased further, and the resource-seeking motive decreased, indicating that the BRI has expanded the importance of market size and labor costs of African countries (regions) while weakening the importance of resource endowments. The influence of institutional quality has become insignificant after 2013. This implies that the BRI, which serves as a crucial mechanism for China-Africa investment cooperation, has somewhat diminished the role of institutional quality in the host countries (regions).

**Table 8:** Effects of BRI.

Model	Before 2013	After 2013	Effect of BRI		
	(1)	(2)	(3)	(4)	(5)
Methods	PPML	PPML	PPML	PPML	Difference GMM
Variables	$OFDI_{it}$	$OFDI_{it}$	$OFDI_{it}$	$OFDI_{it}$	$\ln OFDI_{it}$
$\ln OFDI_{i,t-1}$					0.2069**
					(0.0986)
$\ln GDP_{it}$	0.5196***	0.6461***	0.6132***	0.6280***	3.5624**
	(0.0682)	(0.0685)	(0.0567)	(0.0626)	(1.7612)
$\ln PGDP_{it}$	−0.2311**	−0.4513***	−0.4275***	−0.3621***	−3.9262*
	(0.1141)	(0.0699)	(0.0625)	(0.0685)	(2.0158)
$Res_{it}$	0.1676**	−0.0132	0.0414	0.0297	0.3363
	(0.0778)	(0.0411)	(0.0396)	(0.0516)	(0.2787)
$As_{it}$	0.2617	−0.3406	−0.0449	0.0496	−0.2990
	(0.2085)	(0.4740)	(0.2683)	(0.2877)	(0.1851)
$WGI_{it}$	0.0787***	0.0176	0.0398***	0.0290	0.1294
	(0.0298)	(0.0150)	(0.0152)	(0.0224)	(0.1362)
*BRI*_*it*_			−0.2876	1.2184	10.6455
			(0.2098)	(1.8503)	(7.9842)
$\ln GDP_{it}\times BRI_{it}$				−0.0220	−0.3583
				(0.0812)	(0.2788)
$\ln PGDP_{it}\times BRI_{it}$				−0.1791*	−0.4003
				(0.0968)	(0.2891)
$Res_{it}\times BRI_{it}$				0.0259	0.1533
				(0.0701)	(0.1629)
$As_{it}\times BRI_{it}$				−0.1543	0.7857
				(0.8161)	(2.0408)
$WGI_{it}\times BRI_{it}$				0.0316	0.0659
				(0.0329)	(0.0830)
Cons	−4.6636**	−4.1292**	−3.0760**	−3.7517**	
	(1.9283)	(1.6586)	(1.3868)	(1.5364)	
Control variables	Yes	Yes	Yes	Yes	Yes
Time effect	Yes	Yes	Yes	Yes	Yes
Obs	520	518	1,039	1,039	935
R^2^	0.6756	0.5735	0.5987	0.5989	

The asterisks ***, **, and * indicate levels of signifificance at 1%, 5%, and 10%, respectively. The dynamic model passes AR(1), AR(2) and Sargan tests.

In addition, the BRI is an important policy for investment cooperation between China and African countries, will the BRI facilitate the expansion of Chinese outward FDI in Africa? To verify this conjecture, we further analyse the impact of the BRI on Chinese FDI in Africa. The results are shown in Models (3)-(5). Models (3) include a dummy variable for the time when African countries (regions) join the BRI, and the results show that the BRI itself has an insignificant impact on Chinese FDI in Africa. Model (4) reports the moderating effect of the BRI on investment incentives and institutional quality, the results show that the BRI enhances only the efficiency-seeking motive. A comparison between models (1) and (2) demonstrates that the change in the coefficient of $\ln PGDP_{it}$ is most significant after the year 2013. Model (5) shows that both BRI and the interaction term are not significant under dynamic conditions, reinforcing the conclusion of Model (3).

Combining the results in Table 8, it can be seen that although the BRI is conducive to China-Africa cooperation, and promotes the economic growth of African countries [47], which in turn strengthens to a certain extent the importance of the market size and labor costs in attracting foreign investment. However, the BRI itself has not significantly contributed to the expansion of the scale of Chinese investment in Africa. This is mainly due to the following reasons: Considering the existing development conditions of African countries, China mainly supports African countries with aid under the BRI, and its investment accounts for a relatively low proportion. For example, “The Beijing Action Plan of the Forum on China-Africa Cooperation (2025-2027)” points out that China will provide RMB 360 billion for Africa in the next three years, of which RMB 290 billion will be various kinds of aid and enterprise investment will be no less than RMB 70 billion, which shows that aid occupies an extremely important position in China-Africa cooperation.

However, the results of Models (1) and (2) suggest that the signing of the BRI agreement will help expand the attractiveness of the market size and labor costs in host country (region). Considering the late date of the signing of the agreement between China and African countries, the impact of the BRI can be further analyzed in the future with the deepening of cooperation.

## 6 Conclusion

With the continuous development of China’s industrialisation process, the production capacity of Chinese enterprises has gradually improved, making it possible for enterprises to invest overseas. The transfer of Chinese industries to Africa, accompanied by FDI flows from China to Africa, can not only relieve the supply pressure of China’s surplus capacity but also help African countries (regions) optimise their industrial systems. Accordingly, this study uses data on China’s FDI stock in Africa from 2003 to 2022 to analyse the factors driving China’s investment in Africa from static and dynamic perspectives. This study also analyses the regulatory effect of institutional quality on investment motives and the effect of the BRI.

The findings show that China has clear market-, and efficiency-motives for outward FDI in Africa, but weak resource- and strategic-asset-seeking motives. High institutional quality in the host country (region) is also conducive to expanding China’s outward FDI. The results remain robust after accounting for endogeneity issues, considering geographical factor, excluding special years, applying 1% Winsorization, and controlling country fixed effect. Besides, there is a tipping point for market- and efficiency-seeking motives. Under dynamic conditions, the signs of the coefficients of variables representing investment motives remain unchanged; China has clear market-, efficiency-, and resource-seeking motives in Africa, but no significant strategic-asset-seeking motive. The difference is the resource-seeking motive becomes significant under dynamic conditions. The estimated coefficient of $WGI_{it}$ is no longer significant when using the Difference GMM method. Investment inertia and investment motives play significant roles in the long run.

In addition, institutional quality has a significant regulatory effect on investment motives. With the optimization of the institutional environment in the host country (region), market size becomes more attractive, while labor costs, natural resources, and strategic assets become less. Under dynamic conditions, institutional quality in host countries enhances the market-seeking motive while simultaneously reducing the efficiency-seeking motive. However, there is no statistically significant moderating influence on resource- and strategic asset-seeking motives. These results suggest that market size and labor costs are the primary drivers of China’s investment strategy in Africa in the long run.

The study found that the BRI itself has no significant effect on Chinese investment in Africa yet. While the signing of relevant agreements can, to a certain extent, increase the attractiveness of host country market sizes and labor costs to investment. Based on the above conclusions, we formulate the following policy implications.

First, overall, Chinese FDI in Africa conforms to the general law of foreign investment. The market economy factor of the host country (region) plays a leading role in attracting investments. Moreover, the appeal of African countries’ (regions’) market size and low labor costs has grown after the withdrawal of the BRI. To maximize these advantages, China should focus on investing in labor-intensive industries and essential consumer goods in Africa. This strategy would not only enable the transfer of China’s excess production capacity and optimize capital allocation but also benefit Africa by boosting employment rates and enhancing the consumption levels of its residents.

Second, the quality of host country’s (region’s) institutions is a key factor for Chinese enterprises when considering investments in Africa. High institutional quality can effectively ensure the sustainability and security of investments, and enhancing the positive impact of market-seeking motives. Besides, it can diminish the significance of labor costs to some extent. Therefore, Chinese enterprises should prioritize regions with stronger institutional quality when selecting investment destinations. It can help to explore the host country’s (region’s) market potential through the establishment of long-term and stable cooperative relationships. In particular, when faced with differences in labor costs among potential investment locations, institutional quality becomes a valuable screening criterion. Even if labor costs are higher, Chinese enterprises might choose to invest in regions with better institutional quality, compensating for the lack of labor cost advantages through stable and secure investment partnerships.

Finally, to maximize the potential of the BRI, the Chinese government should prioritize signing relevant agreements with African countries and consistently enhance the terms of investment cooperation. This will help strengthen the investment promotion aspect of the BRI. Notably, China’s resource-seeking motive in Africa has diminished after 2013, indicating that the ‘resources plunderer strategy’ does not underlie Chinese FDI in Africa. The fundamental purpose of Chinese FDI in Africa is to fully utilize the endowment advantages of different countries and jointly promote the economic development of the host country (region) and home country to maximize corporate profits.

## Supporting information

S1 File The original data utilized for the regression analysis. (XLS)
